# Supplementary material for: Leaves, Infusion, and Grounds—A Three–Stage Assessment of Element Content in Yerba Mate (Ilex paraguariensis) Based on the Dynamic Extraction and Mineralization of Residues
Source: Foods. 2024 Feb 6;13(4):509. doi: 10.3390/foods13040509 (PMC10887975; doi:10.3390/foods13040509)
Supplement: Supplementary file 1 [file foods-13-00509-s001.zip › foods-2859823-supplementary.pdf]

Supplementary Material

Leaves, infusion, grounds – a three–stage assessment of element content in yerba mate *(Ilex paraguariensis)* based on a dynamic extraction and mineralization of residues

Anna Różewska, Jędrzej Proch*, Przemysław Niedzielski

Department of Analytical Chemistry, Faculty of Chemistry, Adam Mickiewicz University, Uniwersytetu Poznańskiego 8, 61–614 Poznań, Poland.

***Corresponding author**

mail: jedrzej.proch@amu.edu.pl

phone: +48 61 829 1764

Table S1. Details of samples' characteristics.

Sample No.	Brand Code	Country of Origin	Type (Kind)	Additives
1	A	Argentina	Con palo	–
2	A	Argentina	Con palo	–
3	A	Argentina	Despalada	–
4	B	Paraguay *	Con palo	–
5	B	Paraguay *	Con palo	aromas
6	B	Paraguay *	Despalada	–
7	B	Paraguay *	Con palo	herbs, aromas
8	B	Paraguay *	Con palo	herbs, aromas
9	B	Paraguay *	Con palo	herbs
10	B	Paraguay *	Con palo	fruits, herbs, aromas
11	B	Paraguay *	Con palo	fruits, herbs, aromas
12	C	Brazil *	Despalada	–
13	C	Brazil *	Despalada	fruits, herbs, aromas
14	C	Brazil *	Despalada	herbs, fruits, aromas
15	C	Brazil *	Despalada	flowers, herbs, seeds, aromas
16	C	Brazil *	Despalada	fruits, flowers, aromas
17	C	Brazil *	Despalada	fruits, aromas
18	C	Brazil *	Despalada	herbs, fruits, aromas
19	C	Brazil *	Despalada	herbs, fruit skin, aromas
20	C	Brazil *	Despalada	fruits, flowers, aromas
21	D	Paraguay	Con palo	–
22	D	Paraguay	Con palo	–
23	D	Paraguay	Con palo	–
24	D	Paraguay	Con palo	aromas
25	D	Paraguay	Con palo	herbs
26	E	Brazil *	Despalada	–
27	E	Brazil *	Despalada	fruits, aromas
28	F	Argentina	Con palo	–
29	G	Argentina	Con palo	–
30	H	Argentina	Con palo	aromas

***** – means repacked in Poland and distributed as the Polish trademark.

Table S2. Method quantification limits (MQLs) for microwave–assisted acid mineralization (M) and dynamic water extraction (E) obtained by the ICP OES analysis.

Element	Emission line (nm)	MQL(M) (mg kg^{-1})	MQL(E) (mg kg^{-1})
Al	396.152	1.0	0.5
As	188.980	0.15	0.077
B	249.772	0.068	0.034
Ca	422.673	4.7	2.4
Cd	214.439	0.022	0.011
Co	238.892	0.054	0.027
Cr	267.716	0.040	0.02
Cu	327.395	0.028	0.014
Fe	238.204	0.82	0.41
K	769.897	3.1	1.6
Mg	285.213	2.0	1.0
Mn	257.610	0.55	0.28
Mo	202.032	0.06	0.03
Na	588.995	2.5	1.3
Ni	231.604	0.056	0.028
P	213.618	1.57	0.79
Pb	220.353	0.18	0.090
Rb	780.026	0.12	0.060
S	181.972	6.8	3.4
Se	196.026	0.38	0.19
Si	288.158	0.48	0.24
Sr	460.733	0.054	0.027
Ti	336.122	0.034	0.017
V	292.401	0.084	0.042
Zn	213.857	0.080	0.040

MQL(M) – method quantification limit (microwave–assisted acid mineralization); MQL(E) – method quantification limit (dynamic water extraction).

Table S3. Results obtained by ICP OES after the microwave–assisted acid mineralization of certified reference material (CRM): tea leaves (INCT–TL–1, Poland).

Element	Certified (mg kg^{-1})	Detected (mg kg^{-1})	Recovery (%)
Al	2290±280	2030±217	89±10
As	0.106±0.021	BQL	–
B	26*	20.6±2.9	79±11
Ca	5820±520	6690±545	115±9
Cd	0.03±0.004	0.027±0.004	90±13
Co	0.387±0.042	0.361±0.058	93±15
Cr	1.91±0.22	1.62±0.26	85±14
Cu	20.4±1.5	17.7±1.8	87±9
Fe	432*	397±41	92±10
K	17000±1200	15300±1340	90±8
Mg	2240±170	1990±192	89±9
Mn	1570±110	1380±127	88±8
Mo	x	0.093±0.013	–
Na	24.7±3.2	26.2±3.30	106±13
Ni	6.12±0.52	4.90±0.49	80±8
P	1810*	1520±120	84±7
Pb	1.78±0.24	1.71±0.3	96±17
Rb	81.5±6.5	77.5±8.3	95±10
S	2470±250	2260±230	91±9
Se	0.076*	BQL	–
Si	x	184±26	–
Sr	20.8±1.7	17.4±2.0	84±10
Ti	30*	21.8±3.1	73±10
V	1.97±0.37	1.83±0.35	93±18
Zn	34.7±2.7	29.5±2.5	85±7

U – expanded uncertainty of certified value; SD – standard deviation; * – informative value; BQL – below (method) quantification limit; x – not certified value.

.

Table S4. The results of standard addition method for two samples: dry yerba mate (1) and infusion (2). All added concentrations of standard solutions were presented as content equivalent in sample (mg kg^{-1}).

Element	Sample (1) (mg kg^{-1})	Added (mg kg^{-1})	Found (mg kg^{-1})	Recovery (%)	Sample (2) (mg kg^{-1})	Added (mg kg^{-1})	Found (mg kg^{-1})	Recovery (%)
Al	280	50<br>250	329<br>504	98<br>90	54.9	50<br>250	106<br>295	102<br>96
As	0.43	0.5<br>2.0	1.03<br>2.5	120<br>104	BQL	0.5<br>2.0	0.535<br>1.90	107<br>95
B	30.8	5.0<br>25	34.6<br>53.1	76<br>89	12.8	5.0<br>25	17.2<br>36.6	88<br>95
Ca	8630	500<br>2000	9170<br>10600	108<br>99	515	500<br>2000	930<br>2280	83<br>88
Cd	0.191	0.5<br>2.0	0.736<br>2.44	109<br>112	0.028	0.5<br>2.0	0.438<br>1.77	82<br>87
Co	0.934	1.0<br>5.0	1.86<br>5.96	93<br>101	0.173	1.0<br>5.0	1.02<br>5.02	85<br>97
Cr	0.704	1.0<br>5.0	1.79<br>5.6	109<br>98	0.246	1.0<br>5.0	1.16<br>5.10	91<br>97
Cu	8.62	1.0<br>5.0	9.64<br>13.5	102<br>98	3.19	1.0<br>5.0	4.23<br>8.34	104<br>103
Fe	208	50<br>250	268<br>465	120<br>103	1.77	50<br>250	45.8<br>239	88<br>95
K	12100	500<br>2000	12700<br>14100	120<br>100	5670	500<br>2000	6210<br>7770	108<br>105
Mg	2210	200<br>1000	2380<br>3170	85<br>96	567	200<br>1000	741<br>1480	87<br>91
Mn	2290	200<br>1000	2510<br>3160	110<br>87	410	200<br>1000	614<br>1370	102<br>96
Mo	BQL	0.5<br>2.0	0.39<br>1.61	78<br>81	BQL	0.5<br>2.0	0.365<br>1.62	73<br>81
Na	75.2	25<br>100	98.2<br>165	92<br>90	21.9	25<br>100	50.9<br>126	116<br>104
Ni	4.63	1.0<br>5.0	5.78<br>9.16	115<br>91	2.52	1.0<br>5.0	3.332<br>6.72	81<br>84
P	1040	100<br>500	1140<br>1470	100<br>86	417	100<br>500	524<br>882	107<br>93
Pb	0.22	0.5<br>2.0	0.76<br>2.44	108<br>111	BQL	0.5<br>2.0	0.610<br>1.74	122<br>87
Rb	36.2	5.0<br>25	41.2<br>57.7	100<br>86	14.2	5.0<br>25	19.7<br>36.2	110<br>88
S	753	100<br>500	866<br>1290	113<br>107	421	100<br>500	503<br>891	82<br>94
Se	0.65	0.5<br>2.0	1.04<br>2.54	78<br>95	0.3	0.5<br>2.0	0.89<br>2.46	118<br>108
Si	141	50<br>250	191<br>400	100<br>104	34.5	50<br>250	79.0<br>265	89<br>92
Sr	33.4	5.0<br>25	38.9<br>55.7	110<br>89	2.68	5.0<br>25	7.68<br>26.2	100<br>94
Ti	10.4	1.0<br>5.0	11.2<br>15.1	80<br>94	BQL	1.0<br>5.0	1.01<br>4.85	101<br>97
V	0.416	0.5<br>2.0	0.917<br>2.49	100<br>104	BQL	0.5<br>2.0	0.450<br>1.88	90<br>94
Zn	41.0	5.0<br>25	45.0<br>63.3	80<br>89	12.1	5.0<br>25	16.7<br>36.6	92<br>98

BQL – below (method) quantification limit

Table S5. Total content (mg kg^{-1}) of selected elements in dry yerba mate depending on the origin, kind (type) and composition (purity).

	**Origin**			**Kind (Type)**		**Composition (Purity)**	
	**Argentina (n = 6)**	**Brazil (n = 11)**	**Paraguay (n = 13)**	**Con Palo (n = 17)**	**Despalada (n = 13)**	**Pure Mate (n = 12)**	**With Additives (n =18)**
**Element**	**Median {Range} (AQL)**	**Median {Range} (AQL)**	**Median {Range} (AQL)**	**Median {Range} (AQL)**	**Median {Range} (AQL)**	**Median {Range} (AQL)**	**Median {Range} (AQL)**
**Al**	306 {280−362} (6)	275 {191−346} (11)	280 {223−375} (13)	299 {223−375} (17)	279 {191−346} (13)	297 {324−375} (12)	273 {191−346} (18)
**As**	0.44* (1)	0.43 {0.19−0.58} (8)	0.22 {0.16−0.49} (7)	0.22 {0.16−0.49} (7)	0.42 {0.19−0.58} (9)	0.24 {0.16−0.43} (5)	0.43 {0.17−0.58} (10)
**B**	38.4 {28.8−55.0) (6)	32.9 {26.8−4.74} (11)	37.5 {29.4−48.9} (13)	37.5 {29.4−55.0} (17)	32.9 {26.8−24.8} (13)	35.7 {28.9−51.7} (12)	34.7 {26.8−55.0} (18)
**Ca**	9530 {8630−11200} (6)	9980 {9020–10800} (11)	9770 {9270−10600} (13)	9610 {8630−11200} (17)	9980 {9020−11000} (13)	9690 {8630−10800} (12)	9810 {9270−11200} (18)
**Cd**	0.30 {0.19−0.49} (6)	0.397 {0.362−0.500} (11)	0.44 {0.30−0.56} (13)	0.43 {0.19−5.64} (17)	0.40 {0.28−0.50) (13)	0.40 {0.19−0.56} (12)	0.43 {0.30−0.50} (18)
**Co**	0.75 {0.28−0.93} (6)	0.21 {0.097−0.92} (10)	0.29 {0.13−0.60} (13)	0.33 {0.13−0.93} (17)	0.24 {0.08−0.92} (13)	0.40 {0.23−0.93} (12)	0.28 {0.08−0.92} (18)
**Cr**	0.63 {0.51−0.70} (6)	0.64 {0.52−0.76} (11)	0.54 {0.40−0.79} (13)	0.58 {0.40−0.79} (17)	0.64 {0.53−0.76} (13)	0.61 {0.47−0.76} (12)	0.59 {0.40−0.79} (18)
**Cu**	8.51 {7.26−11.0} (6)	8.57 {6.52−9.65} (11)	7.85 {5.58−9.49} (13)	8.44 {5.58−11.1} (17)	8.01 {6.52−9.65} (13)	7.93 {5.58−9.49} (12)	8.58 {6.34−11.08} (18)
**Fe**	214 {169−312} (6)	149 {92.7−259} (11)	228 {205−297} (13)	233 {169−312} (17)	182 {92.7−259} (13)	221 {169−312} (12)	221 {92.7−290} (18)
**K**	12600 {9900−15300} (6)	11700 {10900−12400} (11)	11500 {10200−12700} (13)	12100 {9900−15300} (17)	11700 {10200−12400} (13)	11900 {9900−15300} (12)	11900 {10200−13100} (18)
**Mg**	2300 {2050−2500} (6)	2500 {2300−2750} (11)	2380 {2080−2910} (13)	2390 {2050−2910} (17)	2490 {2190−2750} (13)	2320 {2050−2700} (12)	2500 {2080−2900} (18)
**Mn**	1930 {1630−2300} (6)	1280 {1170−1940} (11)	1370 {1040−1970} (13)	1450 {1040−2290} (17)	1280 {1170−1940} (13)	1650 {1040−2300} (12)	13400 {1170−1940} (18)
**Mo**	0.10* (1)	0.08 {0.07−0.09} (5)	0.09 {0.08−0.09} (2)	0.09 {0.08−0.10} (3)	0.07 {0.07−0.09} (5)	0.07* (1)	0.08 {0.069−0.10} (7)
**Na**	78.8 {51.6−93.2} (6)	85.0 {76.8 −96.8} (11)	85.9 {72.7−102} (13)	85.9 {51.6−102} (17)	81.9 {72.7−96.7} (13)	81.7 {51.6−96.8} (12)	85.2 {75.0−102} (18)
**Ni**	4.12 {3.10−5.31} (6)	4.17 {3.01−4.39} (11)	4.01 {2.80−5.06} (13)	3.91 {2.80−5.06} (17)	4.18 {3.01−5.31} (13)	3.89 {2.80−5.31} (12)	4.16 {3.10−5.06} (18)
**P**	967 {914−1070} (6)	1040 {899−1220} (11)	1030 {884−1190} (13)	1030 {884−1190} (17)	1040 {899−1220} (13)	1030 {884−1190} (12)	1020 {903−1220} (18)
**Pb**	0.40 {0.22−0.52} (3)	0.45 {0.20−0.62} (7)	0.58 {0.27−0.59} (5)	0.46 {0.22−0.59} (8)	0.41 {0.20−0.62} (6)	0.40 {0.22−0.60} (7)	0.45 {0.20−0.62} (8)
**Rb**	29.7 {25.9−36.2} (6)	35.5 {29.3−43.6} (11)	32.4 {26.0−39.4} (13)	33.4 {25.9−39.4} (17)	34.7 {26.1−43.6} (13)	31.0 {26.1−37.3} (12)	34.0 {25.9−43.6} (18)
**S**	737 {647−838} (6)	761 {591−888} (11)	788 {631−902} (13)	756 {631−902} (17)	761 {701−888} (13)	761 {636−902} (12)	754 {591−888} (18)
**Se**	0.83 {0.65−1.28} (6)	0.94 {0.86−1.23} (10)	0.58 {1.07−0. 39} (11)	0.62 {0.39−1.28} (15)	0.93 {0.54−1.23} (12)	0.70 {0.54−1.07} (12)	0.90 {0.39−1.28} (15)
**Si**	131 {94.1−283} (6)	152 {106−215} (11)	153 {87.3−272} (13)	164 {87.3−283} (17)	134 {94.1−213} (13)	139 {94.1−283} (12)	161 {87.3−272} (18)
**Sr**	35.4 {33.2−42.3} (6)	35.5 {28.2−65.5} (11)	39.2 {23.8−55.4} (13)	38.1 {23.8−55.4} (17)	33.2 {28.2−65.5} (13)	36.3 {29.8−65.5} (12)	38.9 {23.8−54.9} (18)
**Ti**	9.22 {5.52−11.4} (6)	8.92 {4.07−10.2} (11)	10.3 {6.43−14.1} (13)	10.3 {5.52−14.1} (17)	9.09 {4.07−10.5} (13)	9.65 {5.52−13.9} (12)	9.46 {4.07−13.7} (18)
**V**	0.40 {0.24−0.49} (6)	0.32 {0.10−0.40} (11)	0.37 {0.24−0.89} (13)	0.38 {0.24−0.89} (17)	0.33 {0.10−0.49} (13)	0.41 {0.18−0.89} (12)	0.33 {0.10−0.57} (18)
**Zn**	52.8 {27.8−117} (6)	61.0 {48.3−90.4} (11)	82.7 {60.8−105} (13)	80.7 {41.0−117} (17)	61.0 {27.7−105} (13)	68.8 {27.7−105} (12)	73.5 {48.3−117} (18)

AQL — above method qualification limit (the number of results exceeding AQL in round brackets); Range — as content {min–max}.

Table S6. Total content (mg kg^{-1}) of selected elements in yerba mate infusion depending on the origin, kind (type) and composition (purity).

	**Origin**			**Kind (Type)**		**Composition (Purity)**	
	**Argentina (n = 6)**	**Brazil (n = 11)**	**Paraguay (n = 13)**	**Con Palo (n = 17)**	**Despalada (n = 13)**	**Pure Mate (n = 12)**	**With Additives (n =18)**
**Element**	**Median {Range} (AQL)**	**Median {Range} (AQL)**	**Median {Range} (AQL)**	**Median {Range} (AQL)**	**Median {Range} (AQL)**	**Median {Range} (AQL)**	**Median {Range} (AQL)**
**Al**	51.0 {30.2−71.0} (6)	40.7 {13.0−61.3} (11)	38.7 {14.3−56.2} (13)	47.8 {14.3−71.0} (17)	41.0 {13.0−61.3} (13)	42.6 {24.7−71.0} (12)	42.8 {13.0−56.2} (18)
**As**	0.19* (1)	0.23 {0.10−2.86} (7)	0.17 {0.09−0.24} (5)	0.19 {0.09−0.24} (5)	0.23 {0.10−2.86} (8)	0.19 {0.18−0.24} (4)	0.22 {0.10−0.29} (9)
**B**	21.6 {12.8−29.8} (6)	16.2 {5.03−24.5} (11)	20.2 {11.8−22.9} (13)	20.2 {11.8−30.7} (17)	16.2 {5.03−25.4} (13)	18.8 {11.8−25.4} (12)	17.2 {5.03−30.7} (18)
**Ca**	606 {382−1190} (6)	738 {269−1460} (11)	727 {530−1360} (13)	685 {515−1360} (17)	738 {269−1460} (13)	616 {382−950} (12)	938 {269−1460} (18)
**Cd**	0.03 {0.02−0.06} (4)	0.04 {0.02−0.05} (9)	0.04 {0.01−0.06} (13)	0.04 {0.01−0.06} (14)	0.04 {0.02−0.06} (10)	0.04 {0.01−0.06} (9)	0.04 {0.02−0.06} (15)
**Co**	0.20 {0.10−0.38} (6)	0.09 {0.06−0.20} (11)	0.16 {0.10−0.20} (13)	0.14 {0.10−0.38} (17)	0.11 {0.06−0.30} (11)	0.16 {0.10−0.38} (12)	0.13 {0.06−0.20} (16)
**Cr**	0.24 {0.15−0.27} (6)	0.23 {0.12−0.50} (11)	0.18 {0.09−0.35} (13)	0.19 {0.09−0.35} (17)	0.23 {0.12−0.50} (13)	0.23 {0.09−0.34} (12)	0.22 {0.09−0.50} (18)
**Cu**	3.54 {2.74−5.38} (6)	3.96 {2.46−5.13} (11)	3.41 {2.62−4.75} (13)	3.84 {2.62−5.38} (17)	3.80 {2.46−5.13} (13)	3.21 {2.62−4.36} (12)	3.96 {2.46−5.38} (18)
**Fe**	2.32 {1.77−2.81} (6)	2.57 {1.34−3.59} (10)	2.06 {1.45−3.69} (12)	2.28 {1.45−3.69} (16)	2.36 {1.34−3.59} (12)	2.19 {1.51−3.69} (12)	2.34 {1.34−3.59} (18)
**K**	6690 {4010−9550} (6)	7190 {5380−8810} (11)	7080 {4560−8130} (13)	7080 {4560−9550} (17)	7190 {4010−8810} (13)	7050 {4010−9470} (12)	7140 {5380−9550} (18)
**Mg**	730 {466−1340} (6)	723 {428−1040} (11)	725 {425−1430} (13)	857 {425−1430} (17)	723 {428−1040} (13)	802 {466−1040} (12)	714 {425−1340} (18)
**Mn**	444 {303−744} (6)	397 {168−583} (11)	315 {121−467} (13)	320 {121−744} (17)	402 {168−583} (13)	332 {121−744} (12)	376 {136−583} (18)
**Mo**	BQL (0)	BQL (0)	BQL (0)	BQL (0)	BQL (0)	BQL (0)	BQL (0)
**Na**	48.7 {21.9−49.5} (6)	49.7 {43.6−68.5} (11)	47.0 {16.0−78.3} (13)	48.7 {20.8−78.3} (17)	48.0 {16.0−68.5} (13)	46.8 {16.0−68.5} (12)	49.3 {20.8−78.3} (18)
**Ni**	2.46 {1.99−3.09} (6)	2.10 {0.89−2.66} (11)	2.34 {1.20−2.98} (13)	2.34 {1.20−3.09} (17)	2.27 {0.889−2.98} (13)	2.39 {1.19−3.09} (12)	2.27 {0.89−2.92} (18)
**P**	379 {293−491} (6)	529 {402−650} (11)	508 {269−706} (13)	491 {269−706} (17)	497 {340−650} (13)	483 {269−630} (12)	508 {312−706} (18)
**Pb**	BQL (0)	0.10* (1)	0.17* (1)	0.17* (1)	0.10* (1)	0.17* (1)	0.10* (1)
**Rb**	16.0 {13.7−18.8} (6)	19.7 {16.4−26.3} (11)	16.8 {11.7−23.4} (13)	16.6 {11.7−23.4} (17)	19.2 {12.0−26.3} (13)	15.2 {11.7−23.4} (12)	18.6 {14.9−26.3} (18)
**S**	372 {325−428} (6)	338 {224−541} (11)	301 {227−509} (13)	369 {227−509} (17)	331 {224−541} (13)	342 {227−509} (12)	346 {224−541} (18)
**Se**	0.50 {0.30−0.60} (5)	0.43 {0.27−0.54} (8)	0.32 {0.32−0.43} (3)	0.45 {0.30−0.60} (8)	0.40 {0.27−0.54} (8)	0.44 {0.27−0.57} (8)	0.40 {0.32−0.60} (8)
**Si**	40.0 {34.5−58.0} (6)	47.2 {21.2−58.2} (11)	47.9 {28.8−66.2} (13)	44.6 {28.8−66.2} (17)	47.2 {21.2−58.2} (13)	42.5 {28.8−60.4} (12)	46.7 {21.2−66.2} (18)
**Sr**	2.76 {1.90−4.35} (6)	2.57 {1.05−4.00} (11)	3.84 {2.12−8.16} (13)	3.40 {1.90−8.16} (17)	2.83 {1.05−4.00} (13)	2.69 {1.90−4.35} (12)	3.43 {1.05−8.16} (18)
**Ti**	0.02* (1)	0.02 {0.02−0.02} (2)	0.07* (1)	0.02 {0.02−0.07} (2)	0.02 {0.02−0.02} (2)	0.02 {0.02−0.07} (2)	0.02 {0.02−0.02} (2)
**V**	BQL (0)	BQL (0)	BQL (0)	BQL (0)	BQL (0)	BQL (0)	BQL (0)
**Zn**	15.2 {9.18−39.8} (6)	24.1 {13.6−35.9} (11)	26.1 {16.1−36.4} (13)	24.7 {12.1−39.8} (17)	24.1 {9.18−35.9} (13)	21.2 {9.18−35.9} (12)	24.7 {13.6−39.8} (18)

AQL — above method qualification limit (the number of results exceeding AQL in round brackets); Range — as content {min–max}; BQL — below quantification limit (if AQL = 0); * — the value (if AQL = 1);

Table S7. Total content (mg kg^{-1}) of selected elements in yerba mate grounds depending on the origin, kind (type) and composition (purity).

	**Origin**			**Kind (Type)**		**Composition (Purity)**	
	**Argentina (n = 6)**	**Brazil (n = 11)**	**Paraguay (n = 13)**	**Con Palo (n = 17)**	**Despalada (n = 13)**	**Pure Mate (n = 12)**	**With Additives (n =18)**
**Element**	**Median {Range} (AQL)**	**Median {Range} (AQL)**	**Median {Range} (AQL)**	**Median {Range} (AQL)**	**Median {Range} (AQL)**	**Median {Range} (AQL)**	**Median {Range} (AQL)**
**Al**	215 {166−331} (6)	209 {137−275} (11)	221 {149−326} (13)	210 {149−331} (17)	217 {149−326} (13)	205 {149−331} (12)	217 {137−275} (18)
**As**	0.16* (1)	0.18 {0.18−0.34} (2)	0.46* (1)	0.31 {0.16−0.46} (5)	0.26 {0.18−0.34} (2)	0.16* (1)	0.34 {0.18−0.46} (3)
**B**	15.3 {5.62−26.9} (6)	13.6 {6.13−25.9} (10)	17.1 {1.18−28.4} (11)	18.7 {1.18−28.4} (15)	13.6 {6.13−25.9} (12)	11.3 {1.18−26.9} (12)	18.0 {3.92−28.4} (15)
**Ca**	8300 {7510−9710} (6)	8490 {7630−10400} (11)	8470 {7040−9930} (13)	8360 {7040−9930} (17)	8470 {7630−10400} (13)	8300 {7130−9930} (12)	8480 {7030−10400} (18)
**Cd**	0.18 {0.08−0.42} (6)	0.32 {0.17−0.46} (10)	0.29 {0.05−0.43} (13)	0.27 {0.05−0.43} (17)	0.30 {0.08−0.46} (13)	0.27 {0.08−0.43} (11)	0.32 {0.05−0.46} (18)
**Co**	0.11 {0.06−0.21} (5)	0.13 {0.06−0.13} (3)	0.09 {0.07−0.10} (13)	0.10 {0.07−0.10} (6)	0.13 {0.06−0.190 (5)	0.103 {0.06−0.214} (8)	0.07 {0.06−0.13} (3)
**Cr**	0.35 {0.25−0.45} (6)	0.30 {0.15−0.42} (11)	0.30 {0.17−0.41} (13)	0.30 {0.17−0.45} (17)	0.31 {0.15−0.42} (13)	0.31 {0.21−0.45} (12)	0.29 {0.17−0.42} (18)
**Cu**	4.67 {3.45−5.18} (6)	4.04 {2.90−5.31} (11)	3.71 {1.54−5.52} (13)	4.20 {1.54−5.52} (17)	4.04 {2.90−5.31} (13)	4.12 {2.87−5.52} (12)	4.04 {1.53−5.31} (18)
**Fe**	127 {73.6−164} (6)	108 {50.9−201} (11)	188 {90.4−238} (13)	181 {73.6−238} (17)	116 {50.9−201} (13)	155 {73.6−238} (12)	138 {50.9−231} (18)
**K**	4560 {3500−7830} (6)	4460 {1760−5860} (11)	3870 {1940−6490} (13)	3870 {1940−7830} (17)	4460 {1760−6160} (13)	4600 {1940−6940} (12)	3870 {1760−5860} (18)
**Mg**	1640 {1110−1720} (6)	1680 {1570−2010} (11)	1530 {1220−1900} (13)	1570 {1110−1900} (17)	1680 {1570−2010} (13)	1550 {1110−1720} (12)	1670 {1220−2010} (18)
**Mn**	1030 {798−1290} (6)	831 {567−1330} (11)	700 {411−923} (13)	798 {411−1290} (17)	843 {567−1330} (13)	830 {442−1290} (12)	814 {411−1330} (18)
**Mo**	BQL (0)	BQL (0)	BQL (0)	BQL (0)	BQL (0)	BQL (0)	BQL (0)
**Na**	26.9 {17.4−37.3} (6)	25.6 {12.3−34.0} (11)	38.4 {13.4−53.0} (13)	36.0 {13.4−45.5} (17)	25.6{12.3−53.0} (13)	32.3 {14.9−53.0} (12)	26.7 {12.3−45.5} (18)
**Ni**	1.42 {0.77−2.32} (6)	1.15 {0.68−1.77} (11)	0.85 {0.17−1.47} (13)	0.91 {0.17−1.63} (17)	1.21 {0.68−2.32} (13)	1.24 {0.17−2.32} (12)	1.09 {0.47−1.77} (18)
**P**	579 {363−651} (6)	495 {239−603} (11)	459 {241−712} (13)	525 {241−712} (17)	526 {239−603} (13)	547 {239−712} (12)	458 {241−603} (18)
**Pb**	BQL (0)	0.34 {0.28−0.43} (5)	0.33 {0.19−0.48} (4)	0.33 {0.19−0.48} (4)	0.35 {0.28−0.43} (5)	0.39 {0.19−0.48} (3)	0.34 {0.21−0.48} (5)
**Rb**	13.1 {9.66−20.1} (6)	14.1 {9.47−18.9} (11)	19.9 {8.26−23.0} (13)	13.9 {8.26−23.0} (17)	14.2 {9.47−18.9} (13)	14.8 {9.60−23.0} (12)	14.0 {8.26−18.0) (18)
**S**	314 {273−406} (6)	341 {256−456} (11)	391 {279−510} (13)	366 {279−510} (17)	341 {256−533} (13)	389 {273−533} (12)	339 {256−510} (18)
**Se**	0.47 {0.65−0.67} (6)	0.45 {0.43−0.58} (7)	0.57 {0.57−0.71} (3)	0.62 {0.57−0.71} (4)	0.46 {0.43−0.58} (7)	0.67 {0.57−0.71} (3)	0.47 {0.43−0.58) (9)
**Si**	131 {46.7−211} (6)	89.5 {48.0−151} (11)	86.7 {31.1−176} (13)	96.6 {31.1−196} (17)	86.2 {46.7−151} (13)	84.2 {46.7−211} (12)	100 {31.1−196} (18)
**Sr**	30.4 {23.3−38.0} (6)	27.1 {16.1−34.6} (11)	33.3 {17.3−48.2} (13)	33.3 {17.3−48.2} (17)	26.7 {16.1−34.6} (13)	28.9 {23.3−44.9} (12)	30.2 {16.1−48.2} (18)
**Ti**	8.30 {4.15−9.58} (6)	7.60 {3.71−9.60} (11)	8.94 {5.20−13.0} (13)	8.77 {4.79−13.0} (17)	7.60 {3.71−9.60} (13)	8.86 {4.15−13.0} (12)	7.81 {5.20−12.1} (18)
**V**	0.34 {0.13−0.46} (6)	0.24 {0.17−0.38} (9)	0.34 {0.15−0.54} (13)	0.34 {0.13−0.54} (17)	0.25 {0.17−0.46} (11)	0.34 {0.13−0.83} (11)	0.25 {0.15−0.47} (17)
**Zn**	33.9 {16.4−74.1} (6)	31.1 {22.6−55.0} (11)	52.0 {24.8−67.4} (13)	45.5 {24.8−74.2} (17)	31.1 {16.4−61.1} (13)	41.7 {16.4−61.1} (12)	36.7 {22.6−74.1} (18)

AQL—above method qualification limit (the number of results exceeding AQL in round brackets); Range—as content {min–max}; BQL—below quantification limit (if AQL = 0); *—the value (if AQL = 1);

Figure S1. The Spearman's correlation matrix for the content of selected element in dry yerba mate and infusions. Statistically significant coefficients ($p < 0.05$) are bolded.

Infusions \ Dry yerba mate	Al	As	B	Ca	Cd	Co	Cr	Cu	Fe	K	Mg	Mn	Na	Ni	P	Pb	Rb	S	Se	Si	Sr	Ti	V	Zn
**Al**	−0.107	0.103	−0.204	−0.213	−0.102	−0.125	−0.010	0.069	−0.292	−0.135	−0.158	0.220	−0.018	0.160	0.309	−0.026	0.237	−0.255	0.135	−0.022	**−0.369**	0.142	−0.065	−0.193
**As**	0.105	**0.833**	−0.255	0.171	−0.057	**−0.386**	−0.056	0.162	**−0.374**	0.074	0.119	−0.152	−0.161	0.046	0.181	**0.580**	0.216	0.030	0.330	0.124	−0.177	0.138	0.115	−0.138
**B**	0.002	−0.299	**0.491**	−0.147	0.313	0.358	−0.104	−0.127	**0.653**	0.145	−0.059	**0.448**	0.201	−0.122	−0.057	−0.236	−0.226	−0.193	−0.245	−0.107	0.084	0.266	0.093	**0.459**
**Ca**	0.025	0.229	−0.266	0.162	0.002	**−0.486**	−0.255	0.260	−0.195	−0.076	0.101	−0.097	0.147	0.227	0.094	−0.058	−0.036	−0.134	0.099	0.087	−0.228	0.240	0.088	0.049
**Cd**	−0.238	0.159	−0.067	−0.036	**0.481**	−0.334	−0.145	0.001	0.014	−0.064	−0.153	0.133	0.155	−0.036	**0.613**	−0.041	**0.416**	−0.089	−0.042	−0.138	−0.344	**0.390**	−0.014	**0.445**
**Co**	−0.061	−0.232	0.247	**−0.414**	−0.081	**0.627**	0.057	−0.209	0.240	0.079	**−0.499**	**0.605**	−0.131	0.038	0.012	0.022	0.125	−0.142	**−0.480**	−0.227	−0.188	0.132	0.227	0.194
**Cr**	**−0.374**	0.075	−0.250	**−0.374**	−0.075	−0.072	**0.593**	−0.004	−0.235	−0.168	−0.158	0.083	0.100	0.229	**0.504**	0.005	**0.485**	−0.119	−0.184	−0.271	−0.262	−0.024	−0.170	−0.155
**Cu**	−0.030	0.152	−0.089	0.187	0.030	**−0.437**	−0.090	**0.653**	−0.076	0.049	**0.376**	0.134	0.259	0.005	0.182	0.012	0.204	−0.272	−0.001	0.241	−0.138	0.137	0.169	0.178
**Fe**	−0.149	0.257	−0.058	−0.030	0.051	−0.224	0.085	0.267	−0.178	0.162	−0.087	0.072	0.135	−0.112	0.342	**0.371**	**0.415**	−0.307	0.094	0.217	**−0.400**	0.210	0.093	−0.046
**K**	0.019	−0.029	0.349	0.109	0.236	−0.166	−0.120	0.295	0.156	0.325	0.251	0.211	0.113	−0.265	0.112	0.044	−0.014	**−0.429**	0.113	0.256	0.152	−0.048	0.006	0.352
**Mg**	**0.363**	−0.162	0.295	0.115	0.197	−0.021	−0.175	0.096	**0.473**	−0.017	0.321	0.052	0.220	**−0.578**	**−0.376**	0.151	−0.286	−0.107	0.114	0.294	**0.417**	**0.377**	0.340	**0.407**
**Mn**	−0.183	0.163	−0.221	−0.158	−0.145	−0.099	0.349	0.174	−0.342	0.062	−0.155	**0.475**	0.035	0.164	**0.386**	0.058	**0.488**	−0.263	0.097	−0.094	**−0.628**	−0.002	−0.075	−0.112
**Na**	−0.011	0.238	−0.276	0.173	0.104	**−0.406**	0.168	0.131	0.348	0.086	0.091	0.060	**0.693**	−0.093	0.283	0.168	0.255	−0.219	−0.050	−0.003	−0.052	**0.526**	−0.025	0.030
**Ni**	−0.263	0.018	−0.099	−0.143	0.029	0.086	0.223	−0.051	−0.095	−0.042	**−0.374**	**0.487**	0.186	**0.519**	**0.460**	−0.239	0.325	−0.295	**−0.383**	−0.227	**−0.421**	0.154	0.047	0.002
**P**	**−0.543**	0.012	−0.025	−0.358	0.184	−0.136	0.107	−0.318	0.215	−0.221	0.201	−0.064	**0.362**	−0.112	0.351	0.002	0.280	−0.064	−0.262	**−0.362**	0.067	0.057	−0.282	0.252
**Pb**	0.070	0.321	−0.323	0.008	0.029	−0.274	−0.249	−0.247	0.103	−0.278	0.056	−0.209	0.059	−0.164	0.150	0.275	−0.083	0.093	0.326	−0.037	−0.102	0.327	0.096	−0.156
**Rb**	−0.267	0.266	0.133	−0.083	0.203	−0.259	0.159	0.149	−0.205	0.231	**0.459**	−0.029	0.301	−0.163	0.025	0.251	**0.591**	−0.172	0.079	0.079	−0.178	−0.141	−0.310	0.120
**S**	0.137	−0.038	0.046	0.147	−0.307	0.087	0.139	0.014	0.009	−0.052	0.282	0.150	0.069	−0.285	−0.239	0.108	−0.195	**0.483**	0.284	0.301	0.291	−0.071	0.095	−0.068
**Se**	**0.424**	0.211	0.017	0.284	−0.153	−0.218	0.192	**0.609**	−0.254	**0.399**	−0.032	0.226	−0.077	−0.169	−0.050	0.280	0.094	−0.276	**0.659**	**0.396**	−0.219	−0.054	0.099	−0.270
**Si**	−0.010	0.239	0.070	0.142	0.052	−0.220	−0.321	0.176	0.005	0.179	0.179	−0.028	0.272	−0.128	0.063	**0.599**	0.012	−0.051	−0.259	0.333	−0.191	0.270	**0.494**	**0.394**
**Sr**	−0.200	0.019	−0.097	−0.089	−0.088	−0.192	−0.304	0.110	0.055	−0.185	0.087	−0.035	0.177	0.263	0.139	−0.122	−0.148	−0.185	**−0.403**	0.067	0.061	0.299	0.228	0.233
**Ti**	0.231	**0.402**	−0.328	0.033	−0.118	−0.110	0.110	−0.053	−0.153	−0.107	−0.043	−0.047	0.023	−0.003	−0.153	0.235	0.132	0.055	0.154	0.025	−0.172	0.299	0.143	−0.228
**Zn**	−0.309	−0.014	0.290	−0.072	**0.547**	−0.225	−0.248	−0.063	0.236	0.188	−0.008	0.049	0.291	−0.240	**0.389**	0.001	0.329	−0.069	−0.164	−0.079	−0.187	0.321	−0.104	**0.702**

1.0

−1.0

Figure S2. The Spearman's correlation matrix for the content of selected element in dry yerba mate and grounds. Statistically significant coefficients ($p < 0.05$) are bolded.

Dry yerba mate (columns) vs. Grounds (rows); colour scale from 1.0 to –1.0

Grounds	Al	As	B	Ca	Cd	Co	Cr	Cu	Fe	K	Mg	Mn	Na	Ni	P	Pb	Rb	S	Se	Si	Sr	Ti	V	Zn
**Al**	**0.735**	**0.393**	–0.108	**0.632**	0.092	–0.210	–0.010	0.333	0.294	0.052	0.008	0.089	0.050	–0.039	–0.289	0.291	–0.305	–0.278	0.124	0.278	0.169	0.329	**0.487**	0.040
**As**	–0.082	**0.542**	–0.289	0.030	–0.108	–0.242	0.162	0.095	–0.092	–0.051	0.043	–0.025	0.205	**0.364**	0.318	0.173	0.184	–0.279	–0.008	0.112	–0.021	0.210	0.077	–0.226
**B**	0.314	0.067	**0.583**	**0.411**	–0.154	0.126	–0.103	0.360	–0.028	**0.505**	0.283	0.170	0.016	–0.273	**–0.506**	**0.379**	–0.245	–0.067	0.254	**0.753**	0.260	0.018	0.357	0.221
**Ca**	**0.517**	**0.382**	–0.101	**0.851**	0.101	–0.316	–0.044	**0.501**	0.074	0.304	0.071	–0.055	0.083	–0.090	–0.166	0.203	–0.121	–0.193	0.324	0.338	0.051	0.173	0.235	–0.139
**Cd**	0.080	**0.424**	0.057	**0.457**	**0.398**	**–0.398**	**–0.367**	**0.439**	0.031	**0.437**	–0.066	–0.074	0.097	–0.161	0.264	0.306	0.353	–0.139	0.079	0.340	–0.030	**0.385**	0.119	**0.385**
**Co**	0.073	–0.338	0.164	**–0.418**	–0.233	**0.657**	0.231	–0.347	–0.096	–0.128	–0.220	0.260	**–0.590**	0.208	–0.230	–0.253	–0.303	0.080	0.103	–0.264	0.038	**–0.584**	–0.119	–0.322
**Cr**	0.075	0.110	–0.219	0.169	–0.039	0.189	**0.566**	0.066	0.218	–0.279	–0.154	0.331	0.033	0.105	–0.051	–0.257	0.156	0.246	–0.018	–0.212	–0.098	0.156	0.220	–0.181
**Cu**	**0.496**	0.190	0.055	**0.477**	–0.195	–0.091	0.091	**0.725**	0.112	**0.427**	0.004	0.042	0.099	–0.136	–0.324	**0.413**	–0.090	–0.285	0.231	**0.666**	0.180	0.150	**0.410**	–0.075
**Fe**	–0.075	–0.170	0.066	–0.063	0.188	0.194	0.006	–0.018	**0.741**	–0.162	–0.059	0.053	0.323	–0.229	–0.044	–0.086	–0.041	–0.113	**–0.643**	–0.165	**0.384**	**0.467**	0.297	0.357
**K**	0.050	–0.017	0.075	0.131	–0.181	0.277	0.074	0.027	–0.095	0.344	–0.136	–0.045	–0.017	0.034	–0.232	0.289	0.015	0.179	–0.153	0.199	–0.114	–0.028	0.164	–0.152
**Mg**	–0.343	0.297	–0.206	–0.067	–0.214	–0.046	0.309	–0.003	**–0.419**	–0.172	**0.424**	–0.101	–0.046	**0.433**	0.026	–0.086	0.257	0.155	0.060	–0.160	–0.246	**–0.473**	**–0.366**	**–0.406**
**Mn**	0.192	0.047	0.012	0.091	**–0.390**	0.284	**0.381**	**0.379**	–0.229	**0.386**	–0.114	**0.539**	–0.236	0.091	–0.148	0.190	0.170	–0.098	0.172	0.192	–0.279	–0.285	0.040	–0.280
**Na**	–0.097	–0.241	**0.535**	0.003	0.173	0.178	–0.338	–0.302	0.037	0.232	–0.016	–0.145	0.063	–0.292	–0.170	0.140	–0.244	0.155	–0.220	0.151	0.145	–0.014	0.118	**0.428**
**Ni**	0.122	0.122	–0.071	0.086	–0.238	0.303	0.280	–0.023	**–0.420**	0.103	–0.138	–0.021	**–0.467**	**0.442**	–0.153	–0.056	–0.047	0.254	0.156	0.017	–0.120	–0.306	–0.136	**–0.468**
**P**	0.131	0.101	–0.059	0.260	0.076	0.101	–0.032	0.266	–0.046	0.241	–0.298	0.071	–0.225	0.091	0.339	0.150	–0.021	0.119	–0.007	0.222	–0.167	0.154	0.309	–0.073
**Pb**	–0.009	0.348	–0.066	0.035	–0.127	–0.334	–0.209	0.179	–0.095	0.140	0.220	–0.358	0.224	–0.267	0.139	**0.804**	0.029	–0.070	–0.045	**0.413**	0.123	0.294	0.333	0.208
**Rb**	–0.191	0.157	–0.338	–0.211	0.060	–0.049	0.058	–0.048	–0.196	0.051	**–0.590**	0.065	–0.180	0.315	**0.549**	–0.121	**0.475**	0.132	–0.156	–0.342	**–0.479**	0.071	–0.149	–0.123
**S**	–0.354	0.148	–0.002	–0.341	0.262	0.045	–0.169	**–0.411**	–0.117	–0.131	–0.231	–0.316	–0.199	0.240	**0.375**	–0.033	0.211	0.353	–0.172	**–0.438**	–0.355	0.043	–0.175	0.143
**Se**	**0.402**	0.288	**–0.396**	0.358	–0.205	**–0.514**	–0.239	0.143	–0.288	–0.222	0.205	–0.312	–0.048	–0.117	–0.062	0.152	–0.244	0.175	**0.644**	0.226	0.022	0.161	–0.035	–0.315
**Si**	0.253	0.048	0.320	0.349	–0.268	–0.192	–0.186	**0.581**	–0.107	**0.482**	0.301	–0.027	0.165	–0.351	–0.269	**0.476**	–0.182	–0.124	0.357	**0.950**	0.263	0.072	**0.367**	0.082
**Sr**	**0.448**	–0.090	0.331	0.233	0.059	–0.038	–0.067	0.172	**0.422**	0.191	0.051	–0.126	0.233	–0.302	–0.257	0.281	**–0.369**	–0.184	–0.041	**0.589**	**0.754**	0.322	0.300	0.335
**Ti**	0.144	0.303	–0.037	0.117	0.213	–0.265	–0.116	0.160	**0.466**	–0.058	–0.060	–0.077	**0.500**	**–0.391**	0.178	**0.431**	0.046	–0.062	–0.180	0.206	0.119	**0.883**	**0.567**	**0.376**
**V**	**0.377**	–0.010	–0.006	**0.443**	–0.051	–0.176	–0.149	**0.449**	0.320	–0.020	–0.022	0.083	0.246	**–0.374**	–0.196	0.356	–0.222	–0.156	–0.141	**0.442**	0.107	**0.406**	**0.834**	0.327
**Zn**	0.034	–0.142	**0.638**	0.148	**0.384**	0.140	–0.318	0.081	**0.518**	0.354	–0.027	0.140	0.159	**–0.469**	0.004	0.162	–0.058	–0.030	–0.286	**0.401**	**0.463**	**0.390**	0.350	**0.838**

Figure S3. The Spearman's correlation matrix for the content of selected element in yerba mate grounds and infusions. Statistically significant coefficients ($p < 0.05$) are bolded.

Infusions \ Grounds	Al	As	B	Ca	Cd	Co	Cr	Cu	Fe	K	Mg	Mn	Na	Ni	P	Pb	Rb	S	Se	Si	Sr	Ti	V	Zn
**Al**	–0.236	0.309	–0.044	–0.180	0.050	–0.116	–0.152	–0.162	–0.189	–0.312	0.212	0.025	–0.275	–0.078	–0.063	–0.116	–0.014	0.031	0.240	0.018	–0.282	0.034	–0.199	–0.302
**As**	0.335	0.233	0.207	0.304	**0.434**	–0.327	–0.079	0.162	–0.282	0.075	0.236	0.153	–0.157	0.186	0.087	**0.441**	0.116	0.151	0.338	0.148	–0.103	0.191	0.008	–0.169
**B**	–0.014	–0.026	–0.003	–0.189	–0.132	0.053	–0.012	–0.135	**0.455**	**–0.401**	–0.289	–0.212	0.123	**–0.521**	–0.277	**–0.365**	–0.109	0.036	**–0.417**	–0.114	0.001	0.259	–0.010	0.308
**Ca**	0.176	0.176	–0.035	–0.042	0.200	**–0.467**	–0.005	–0.017	–0.157	–0.144	0.016	–0.121	–0.193	–0.136	–0.147	0.052	0.049	0.034	0.300	0.070	–0.172	0.172	0.063	–0.176
**Cd**	–0.071	0.119	–0.258	–0.176	0.331	–0.352	–0.025	–0.296	–0.067	**–0.400**	–0.180	–0.294	–0.062	**–0.395**	0.027	0.023	0.194	0.149	0.079	–0.184	–0.111	0.309	0.045	0.203
**Co**	–0.075	–0.122	0.022	–0.238	–0.119	0.215	0.099	–0.107	**0.403**	0.103	–0.079	0.277	0.068	–0.010	–0.007	–0.154	0.097	0.158	**–0.501**	–0.306	–0.149	0.060	0.075	0.091
**Cr**	–0.259	0.100	–0.260	–0.244	–0.108	–0.060	0.099	–0.151	0.031	–0.063	0.311	0.212	–0.325	0.093	–0.001	0.042	0.251	0.186	–0.231	–0.284	–0.261	–0.021	–0.180	–0.332
**Cu**	0.055	0.125	0.054	0.090	0.192	**–0.442**	–0.044	0.090	0.064	–0.254	0.172	0.034	–0.228	–0.351	0.005	0.107	–0.125	–0.172	0.065	0.215	–0.149	0.220	0.305	–0.002
**Fe**	–0.042	0.279	0.207	0.032	0.307	**–0.375**	–0.067	0.160	–0.043	0.037	0.323	0.145	–0.147	–0.043	0.037	0.242	–0.003	0.143	0.029	0.210	–0.216	0.272	0.059	–0.085
**K**	–0.028	0.077	0.179	0.089	0.118	0.009	**–0.446**	0.026	0.028	**–0.670**	–0.214	–0.149	0.105	**–0.613**	–0.178	0.002	–0.236	–0.298	–0.144	0.326	0.133	0.021	0.053	**0.365**
**Mg**	0.187	–0.282	0.284	0.013	0.011	0.014	–0.119	0.051	0.180	**–0.381**	**–0.621**	–0.280	0.180	**–0.489**	–0.212	0.102	**–0.483**	**–0.392**	0.122	0.328	**0.461**	0.349	**0.385**	**0.501**
**Mn**	–0.144	0.323	–0.116	–0.104	0.090	–0.070	0.162	–0.036	–0.289	–0.115	0.292	**0.465**	**–0.434**	0.059	0.102	–0.061	0.243	–0.010	0.016	–0.098	**–0.491**	–0.068	–0.129	–0.310
**Na**	0.176	0.345	–0.283	0.218	0.273	**–0.541**	0.180	0.167	0.245	–0.090	–0.059	0.006	**–0.489**	–0.315	–0.105	0.066	0.232	–0.116	0.066	–0.058	0.016	**0.393**	–0.022	–0.113
**Ni**	–0.072	**0.376**	–0.229	–0.147	–0.114	–0.014	0.164	–0.228	0.065	–0.159	0.119	0.091	–0.126	–0.039	0.085	–0.249	0.289	0.084	–0.327	–0.331	–0.274	0.051	–0.091	–0.174
**P**	–0.348	0.115	–0.315	**–0.435**	–0.060	–0.201	–0.217	–0.358	**0.374**	**–0.465**	0.209	–0.361	–0.105	**–0.625**	**–0.634**	0.128	–0.104	0.147	–0.358	–0.293	–0.134	0.057	–0.249	0.030
**Pb**	0.084	0.287	–0.142	–0.022	0.184	–0.196	–0.073	–0.157	–0.066	–0.297	–0.041	–0.310	–0.311	–0.268	–0.202	0.141	–0.042	0.137	**0.455**	0.009	–0.092	0.235	–0.070	–0.138
**Rb**	–0.250	0.122	0.214	–0.009	0.271	–0.290	–0.219	0.038	–0.061	–0.128	**0.434**	0.119	–0.040	–0.231	**–0.440**	0.187	–0.290	–0.019	–0.197	0.153	–0.201	–0.112	–0.231	–0.046
**S**	0.066	–0.118	0.245	–0.028	–0.077	0.152	0.285	0.164	–0.234	0.233	–0.032	0.253	–0.066	0.210	0.184	–0.001	–0.192	**–0.556**	0.276	**0.366**	0.313	–0.108	0.101	0.124
**Se**	0.157	0.005	0.269	0.339	0.130	–0.012	0.050	**0.474**	**–0.438**	0.068	–0.049	**0.426**	–0.236	0.107	0.208	–0.006	0.053	–0.265	0.272	**0.431**	–0.027	0.062	0.101	–0.249
**Si**	0.350	0.012	0.354	0.131	0.318	**–0.435**	–0.224	0.139	0.132	0.147	–0.040	0.035	0.333	–0.188	0.050	**0.659**	–0.206	0.117	–0.075	0.215	0.075	0.344	**0.491**	0.271
**Sr**	–0.026	0.308	–0.032	–0.229	0.006	**–0.378**	–0.176	–0.197	0.306	–0.269	0.055	–0.331	0.038	–0.291	–0.185	0.114	–0.148	0.150	–0.012	0.018	0.002	0.240	0.102	0.078
**Ti**	0.166	**0.364**	0.018	0.079	0.072	–0.105	0.240	0.174	–0.116	0.227	0.060	0.178	–0.122	0.238	0.143	0.139	0.059	–0.082	0.285	–0.006	–0.043	0.172	–0.094	–0.188
**Zn**	–0.130	–0.063	–0.042	–0.189	**0.437**	**–0.540**	–0.237	–0.240	0.220	–0.319	–0.092	–0.312	0.118	**–0.544**	–0.275	0.091	–0.024	0.331	–0.156	–0.126	–0.079	0.257	0.004	**0.374**

1.0

–1.0
